# Neuronally Derived Extracellular Vesicles’ Oligomeric and p129-α-Synuclein Levels for Differentiation of Parkinson’s Disease from Essential Tremor

**DOI:** 10.3390/ijms26083819

**Published:** 2025-04-17

**Authors:** Costanza Maria Cristiani, Selena Mimmi, Elvira Immacolata Parrotta, Mariagrazia Talarico, Anna Maria Tolomeo, Elisabetta Pingitore, Khushboo Fatima, Basilio Vescio, Luana Scaramuzzino, Valentina Crapella, Anna Maria Zimbo, Enrico Iaccino, Giovanni Cuda, Aldo Quattrone, Andrea Quattrone

**Affiliations:** 1 Neuroscience Research Center, Department of Medical and Surgical Sciences, University “Magna Graecia”, 88100 Catanzaro, Italy; 2 Laboratory of Stem Cells, Department of Medical and Surgical Sciences, University “Magna Graecia”, 88100 Catanzaro, Italy; 3 Institute of Pediatric Research Città della Speranza, 35128 Padua, Italy; 4 Department of Cardiac, Thoracic and Vascular Science and Public Health, University of Padova, 35128 Padua, Italy; 5 Department of Experimental and Clinical Medicine, University “Magna Graecia”, 88100 Catanzaro, Italy; 6 Biotecnomed S.c.ar.l., 88100 Catanzaro, Italy; 7 IBSBC-CNR, Via T. Campanella, 115, 88100 Catanzaro, Italy; 8 Institute of Neurology, Department of Medical and Surgical Sciences, University “Magna Graecia”, 88100 Catanzaro, Italy

**Keywords:** Parkinson’s disease, essential tremor, neuronally derived extracellular vesicles, oligomeric α-synuclein, p129-α-synuclein

## Abstract

Clinical differentiation between Parkinson’s disease (PD) and essential tremor (ET) may be challenging, highlighting the need for easily assessable diagnostic biomarkers. Neuronally derived extracellular vesicles (NDEVs) have been proposed as a peripheral matrix that can well recapitulate the cellular composition of neurons. We investigated the clinical usefulness of NDEV oligomeric and p129-α-synuclein levels in discriminating between patients with PD and those with ET. NDEV oligomeric and p129-α-synuclein species were assessed using an ELISA in 43 patients with PD, 21 patients with ET, and 45 healthy controls (HCs). NDEV oligomeric α-synuclein levels were significantly higher in PD in comparison with ET and HCs, while p129-α-synuclein values were significantly lower in HCs compared to other groups. By using a receiver operator characteristic (ROC) analysis, oligomeric-α-synuclein achieved an excellent classification performance in distinguishing PD from both ET and HCs (AUC: 0.976 and 0.997, respectively), while lower performance was obtained in differentiating ET from HCs (AUC: 0.85). On the other hand, p129-α-synuclein accurately discriminated both PD and ET from HCs (AUC: 0.997 and 0.952, respectively) but had very low performance in differentiating PD from ET (AUC: 0.47). Our study suggests that NDEV oligomeric α-synuclein is an accurate blood-derived biomarker to differentiate PD from ET, while p129-α-synuclein may be useful in distinguishing ET from HCs.

## 1. Introduction

Parkinson’s disease (PD) and essential tremor (ET) represent the most common tremor disorders in the elderly [1]. Clinically, PD is a neurodegenerative disease characterized by the presence of unilateral rest tremor together with a wide range of additional motor symptoms, such as bradykinesia, gait impairment, and rigidity [2]. On the other hand, ET is characterized by bilateral action tremor of the upper limbs with or without tremor in other parts of the body and without other overt neurological signs [3]. However, ET is phenotypically heterogenous, with a high proportion of patients displaying additional symptoms such as resting tremor, subtle dystonic features, gait impairment, and other mild neurological signs of uncertain significance, collectively termed as ET-plus [3,4]. Moreover, action tremor, which represents the core symptom in ET, can be commonly observed in tremulous patients with PD [1], overall complicating differential diagnosis. Single-photon emission computed tomography with 123I-ioflupane (DaTscan) has been considered as the gold standard technique for differentiating PD from ET [5,6], but it is an expensive, invasive, and time-consuming nuclear medicine procedure. Easily accessible biomarkers for differentiating PD from ET would be highly valuable in clinical practice for the proper management of patients and for their correct inclusion in clinical trials. Neurophysiological assessments of tremor have shown potential to support differential diagnosis [7,8]. However, biomarkers reflecting the molecular bases of the diseases have been gaining interest and may play a significant role in differential diagnosis.

At the molecular level, the pathological hallmark of PD is the widespread presence within the brain of protein deposits known as Lewy bodies (LBs), composed of aggregated α-synuclein [9]. Physiologically, α-synuclein is a monomeric unfolded protein of presynaptic terminals. However, following environmental cues, α-synuclein can change its native conformation to form soluble oligomers, which then precipitate into insoluble fibrils to form LBs [9,10]. Notably, aggregated α-synuclein within LBs has been found to be phosphorylated at Ser129 (p129-α-synuclein) [11], although the pathological role of such post-translational modification is still debated [12]. The pivotal importance of α-synuclein has prompted investigations in accessible blood-derived fluids regarding its effectiveness as a biomarker to distinguish PD from healthy subjects and from other movement disorders. However, measurements in plasma or serum have provided inconsistent results so far [13,14,15,16], possibly due to α-synuclein release from disrupted red blood cells, which represent the most abundant peripheral source of this protein [17,18].

Extracellular vesicles (EVs) are small vesicles of 40–180 nm that are virtually released by all cell types and contain biomolecules reflecting the state and composition of the parent cell. Blood-derived EVs have been proposed as a more reliable source of biomarkers compared to the serum/plasma itself [19]. Moreover, proteins encapsulated within neuronal-derived EVs (NDEVs) have been suggested to provide a more precise “window” on pathological processes occurring within the central nervous system (CSN) [20]. Notably, NDEVs can cross the blood–brain barrier and be easily isolated in blood by immunocapture, employing an antibody against the neuronal protein L1CAM [21]. Previous studies have demonstrated that NDEV α-synuclein distinguished PD from parkinsonism [22,23,24,25,26,27], while no difference was found in NDEV α-synuclein levels between tremulous PD and ET [28]. Here, we investigated whether oligomeric and p129-α-synuclein species, specifically associated with LBs, could be detected in NDEVs and could be used as potential biomarkers to discriminate between PD and ET in clinical practice.

## 2. Results

### 2.1. Demographic and Clinical Features

The study cohort included 43 patients with PD and 21 patients with ET as well as 45 healthy controls (HCs), whose demographics and clinical characteristics are summarized in Table 1. While PD and ET showed similar sex distribution, females were predominant in the HC group. Patients with ET were younger and had longer disease duration compared to those with PD, according to the earlier onset of the disease [3].

### 2.2. Oligomeric and p129-α-Synuclein in NDEVs

**Figure 1:**
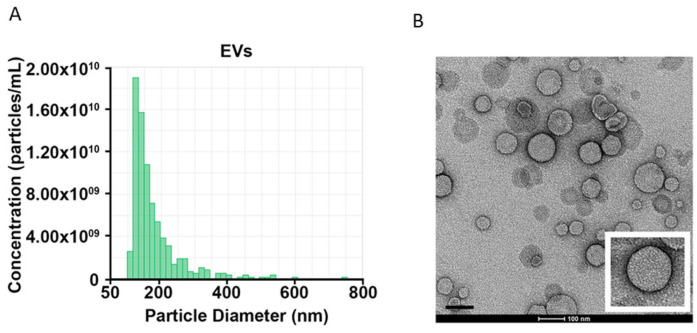
Physical characterization of EVs. EV size distribution was analyzed using tunable resistive pulse sensing (tRPS) technology (**A**) and transmission electron microscopy (**B**) (scale bar = 100 nm; insert shows higher-magnification image). Both approaches confirmed that isolated vesicles could be referred to as “small EVs”.

Nanoparticles from our subjects were characterized by morphological assessment. They showed a spherical shape with a diameter of 30–150 nm as well as the presence of a lipid bilayer, thus largely fulfilling the MISEV2018 criteria for “small EVs” (Figure 1A,B) [29]. From the whole small EV mixture, NDEVs were isolated based on the expression of the neuronal surface marker L1CAM and lysed, and α-synuclein species were quantified using an ELISA assay. In order to normalize NDEV number and protein content among subjects, NEDVs were isolated from the same starting serum amount and lysed in the same volume of lysis buffer (500 µL for both).

Oligomeric and p129-α-synuclein species were detectable in NDEVs from all three groups. Since the PD and ET groups showed significant differences in sex distribution, age, and disease duration (*p*-value < 0.05), comparisons of NDEV oligomeric and p129-α-synuclein levels in the patient groups were corrected for these three variables, while comparisons among the patients with ET or PD and HCs were corrected for age and sex.

**Figure 2:**
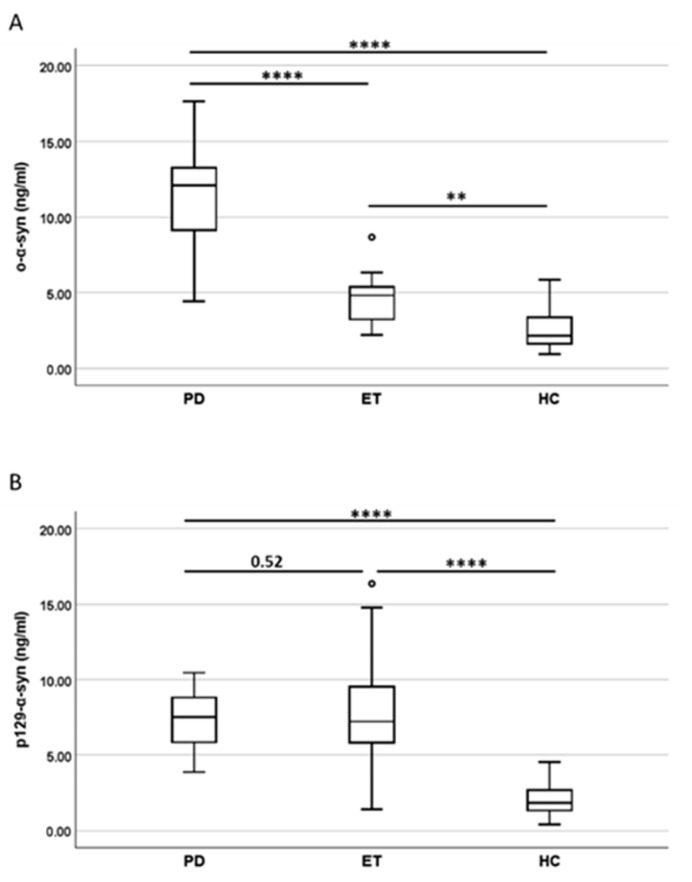
NDEV concentrations of o-α-synuclein (**A**) and p129-α-synuclein (**B**) in PD (*n* = 43), ET (*n* = 21), and HC (*n* = 45). Data are summarized as box plots, where upper, middle, and lower lines represent 75th percentile, median, and 25th percentile, respectively. Limits of vertical lines represent ranges, while dots represent moderate outliers. Statistical analysis was performed using ANCOVA, with sex, age (HC vs. PD vs. ET), and disease duration (PD vs. ET) as covariates, followed by Tukey’s HSD test (****: *p*-value < 0.0001; **: *p*-value < 0.01). o-α-syn = oligomeric α-synuclein; p129-α-syn = 129Ser-phosphorylated α-synuclein; PD = Parkinson’s disease; ET = essential tremor; HC = healthy control.

The NDEV oligomeric α-synuclein levels were significantly higher in PD compared to the other groups (*p* < 0.0001 in both cases) and also significantly higher in ET compared to HCs (*p* = 0.0044) (Figure 2A). On the other hand, NDEV p129-α-synuclein levels were significantly higher in both patient groups compared to HCs (*p* < 0.0001 in both cases), with similar values between patients with PD and those with ET (*p* = 0.52) (Figure 2B).

No significant correlations were detected between levels of NDEV α-synuclein species and demographical or clinical features in our patient groups (Table 2).

### 2.3. Classification Performances Between Groups

**Figure 3:**
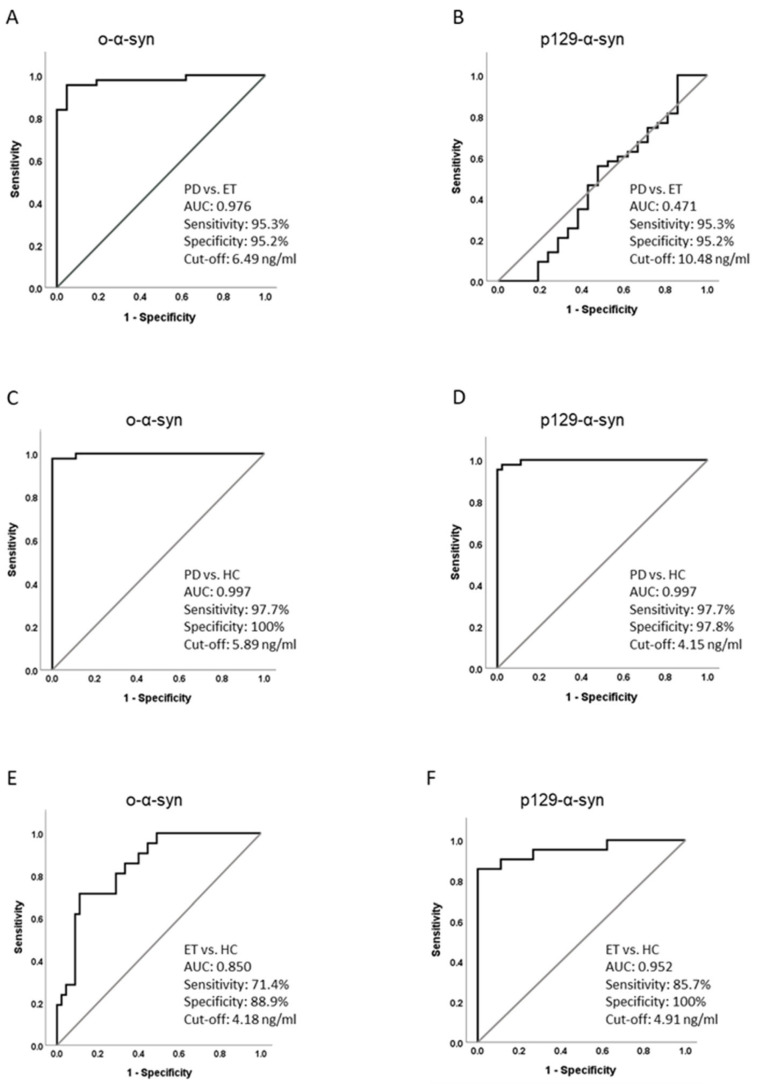
Receiver operating characteristic curves for o-α-synuclein (**A**,**C**,**E**) in differentiating PD from ET (**A**), PD from HC (**C**), and ET from HC (**E**) and for p129-α-synuclein (**B**,**D**,**F**) in differentiating PD from ET (**B**), PD from HC (**D**), and ET from HC (**F**). o-α-syn = oligomeric α-synuclein; p129-α-syn = 129Ser-phosphorylated α-synuclein; PD = Parkinson’s disease; ET = essential tremor; HC = healthy control; AUC = area under the curve.

To further evaluate the accuracy of NDEV synuclein species in discriminating between groups, receiver operating characteristic (ROC) curves were employed. NDEV oligomeric α-synuclein levels showed excellent performance in distinguishing PD from both ET and HC (PD vs. ET: AUC = 0.976, 95% CI: 0.942–1.00; PD vs. HC: AUC = 0.997, 95% CI: 0.992–1.00) (Figure 3A,C), while lower performance was obtained in comparing ET with HC (AUC = 0.85, 95% CI: 0.756–0.942) (Figure 3E). In particular, when a cut-off of 6.49 ng/mL was used, NDEV oligomeric α-synuclein values distinguished PD from ET with a sensitivity of 95.3% (95% CI: 84.54–99.17%) and a specificity of 95.2% (95% CI: 77.33–99.76%) (Figure 3A), and a similar cut-off (6.69 ng/mL) optimally discriminated PD from HC with a sensitivity of 97.7% (95% CI: 87.94–99.88%) and a specificity of 100% (95% CI: 92.13–100%) (Figure 3C).

On the other hand, NDEV p129-synuclein showed similar values between PD and ET and had an AUC of 0.47 (95% CI: 0.307–0.634) in this comparison (Figure 3B), but it was highly accurate in discriminating each patient group from HCs, with a slightly better performance for PD (AUC = 0.997, 95% CI: 0.991–1.00) than for ET and HC (AUC = 0.952, 95% CI: 0.889–1.00) (Figure 3D,F). In detail, when a cut-off of 4,15 ng/mL was used, NDEV p129-α-synuclein values distinguished PD from HC with a sensitivity of 97.7% (95% CI: 87.94–99.88%) and a specificity of 97.8% (95% CI: 88.43–99.89%) (Figure 3D), and a similar cut-off of 4.91 ng/mL discriminated ET from HC with a sensitivity of 85.7% (95% CI: 65.36–95.02%) and a specificity of 100% (95% CI: 92.13–100%) (Figure 3F).

## 3. Discussion

In the current study, we demonstrated that oligomeric and p129-α-synuclein were encapsulated in different amounts within NDEVs from patients affected by PD or ET compared to healthy subjects. Particularly, we showed that oligomeric α-synuclein levels were more abundant in NDEVs from patients with PD and that such a biomarker accurately distinguished PD from ET and HC. In addition, we also demonstrated that NDEVs from subjects affected by either PD or ET contained a higher amount of p129-α-synuclein compared to HCs, allowing for excellent discrimination of both patient groups from HCs.

Currently, the differential clinical diagnosis of ET or PD relies on clinical symptoms, since the two diseases show different types of tremors [2,3]. However, resting tremor, one of the key signs of PD, as well as other neurological signs can be frequently detected in ET, making clinical misdiagnoses common [30] and highlighting the need for biomarkers to be used in clinical practice. Molecular biomarkers within peripheral biofluids are highly desirable since they are easily accessible and may also provide insights into the underlying molecular disease bases. Accordingly, α-synuclein species have been widely investigated in different matrices as biomarkers for PD and other synucleinopathies, but the results are conflicting and overall inconclusive [13,14,15,16], possibly due to contamination of α-synuclein by disrupted RBCs [17,18]. The only molecular assay with sufficiently high sensitivity and specificity in establishing α-synuclein pathology is the detection of misfolded α-synuclein by real-time quaking-induced conversion (RT-QuIC), which can only be applied to cerebrospinal fluid and skin [31]. However, lumbar puncture is an invasive procedure that is poorly scalable in clinical routine, while skin results may be largely affected by biopsy depth and position. Conversely, the method of assessing α-synuclein using RT-QuIC in plasma or serum is still under investigation, and more evidence is needed to endorse the applicability of this method [31].

In this context, NDEVs, encapsulating proteins directly deriving from affected neurons, may represent a reliable substrate to dose α-synuclein. Most existing studies in parkinsonism focused on total α-synuclein [22,23,24,25,32], but promising evidence also emerged for oligomeric [26] and p129-α-synuclein levels [22,27]. Notably, both NDEV total and oligomeric α-synuclein levels were higher in PD compared to other neurodegenerative parkinsonian syndromes without α-synuclein pathology, such as progressive nuclear palsy and corticobasal syndrome [22,23,24,26], while differences in total and p129-α-synuclein levels between PD and other synucleinopathies, such as multiple system atrophy and dementia with Lewy bodies, were less consistent [22,23,24,25,27]. In our study, NDEVs oligomeric α-synuclein levels appeared to be PD-specific, being markedly elevated in these patients and accurately differentiating PD from ET with an AUC of 0.976 and controls with an AUC of 0.997, respectively. This result is in accordance with the pathological role attributed to α-synuclein oligomers in PD [9,10].

Although ET is not considered a synucleinopathy, LBs have been detected in 25% of patients with ET [33]. Moreover, altered levels of α-synuclein have been found within erythrocytes from patients with ET [34], suggesting that alteration(s) in α-synuclein regulation might occur in ET. Our results are in line with these findings, showing that NDEV p129-α-synuclein levels were higher in PD and ET than in controls. Moreover, our study demonstrates that p129-α-synuclein accurately differentiated ET from controls (AUC: 0.952; specificity 100%), suggesting its usefulness in differentiating these two participant groups. Notably, there is no consensus about the function of such a post-translational modification in LB formation. While its abundance in LBs suggested a pathological role [12], a more recent study demonstrated that phosphorylation at Ser129 attenuated α-synuclein propension to aggregate and related cytotoxicity [35]. Therefore, in both PD and ET, the increased values of p129-α-synuclein in NDEVs might reflect an active attempt by neurons to limit fibril deposition and LB formation. The role of α-synuclein in ET is currently poorly investigated, and further research is warranted to clarify this point.

Overall, this is the first study investigating oligomeric and p129-α-synuclein levels in NDEVs from patients affected by essential tremor and comparing LB-related α-synuclein species in NDEVs from patients with PD and patients with ET. Our evidence suggests that NDEV oligomeric α-synuclein might be a highly accurate and accessible biomarker to differentiate PD from ET and HC, whereas high p129-α-synuclein levels are less specific for PD and can be observed in ET as well, also holding promise for accurate differentiation between patients with ET and HCs in clinical practice. Our study has several strengths. First, we investigated oligomeric and p129-α-synuclein in distinguishing among PD, ET, and HC, allowing us to compare these two key α-synuclein species. Second, all patients underwent DaTscan, which is considered the gold standard procedure to discriminate PD from ET, drastically reducing the risk of misdiagnosis between these two diseases in our cohort.

Our study also has some limitations. First, our ET cohort was small and did not allow subgroup stratification. Studies in larger patient cohorts are needed to confirm our findings. Second, post-mortem neuropathological diagnostic confirmation is missing. Although PD diagnosis was performed by movement disorder specialists with more than 10 years of experience based on international criteria [2] and supported by DaTscan, it is still possible that some misdiagnoses with other parkinsonisms characterized by nigrostriatal degeneration might have occurred [36] in a few patients. Of note, we isolated NDEVs based on the expression of L1CAM, whose utility has been recently questioned [37,38]. Indeed, L1CAM expression is not restricted to neurons within CNS but is also detected in peripheral neurons as well as in many types of non-neuronal cells [37]. Moreover, L1CAM has been shown to exist in multiple forms, both membrane-bound and soluble, which compromises its specificity [38]. Therefore, L1CAM-based isolation of EV is expected to enrich NDEVs from CNS rather than to provide a pure population. As a consequence, cargoes from EVs not originating from CSN might have contributed to the observed results. In the absence of more specific markers, L1CAM represents the most shared choice to isolate NDEVs [37]. Whether additional markers and/or purification strategies are developed, it will be interesting to confirm the results obtained on enriched NDEVs on purer populations.

## 4. Materials and Methods

### 4.1. Participants

A total of 21 patients with ET, including 13 with and 8 without resting tremor, along with 43 patients with PD all fulfilling MDS diagnostic criteria [2,3], were enrolled. For all patients, a neurological examination was performed by movement disorder specialists. Moreover, patients underwent a 3T brain MRI and DaTscan. Rest tremor in ET was further confirmed by surface electromyographic assessment. For patients with PD, the neurological examination included the MDS—Unified Parkinson’s Disease Rating Scale and Hoehn and Yahr (HY) rating scales in practical off state to score disease severity, while the Fahn–Tolosa–Marín tremor rating scale was used for patients with ET. For both patient groups, the exclusion criteria included clinical features and MRI abnormalities suggestive of other diseases, diffuse brain vascular lesions or lacunar infarctions in the basal ganglia, and current or past use of medications known to cause or exacerbate tremor. As HCs, 45 subjects without any neurological disorders as well as without close relatives affected by neurodegenerative diseases were recruited. The study was performed in accordance with the Declaration of Helsinki and approved by the Calabria Region Ethics Committee. Written informed consent for study participation and usage of medical records for research purposes was obtained from all participants.

### 4.2. EV Characterization, NDEV Isolation, and α-Synuclein Species Quantification

Serum samples from each enrolled patient were collected between 9 a.m. and 12 p.m. in BD Vacutainer™ SSTTM Serum Separation Tubes (BD, Franklin Lakes, NJ, USA), processed within 30 min by centrifugation at 3000 rpm for 10 min at 20 °C, aliquoted, and stored at −80 °C until use.

EV isolation was performed by two-step size exclusion chromatography, as previously described [39]. Briefly, 500 μL of serum was placed into qEVoriginal/35 nm columns (Izon Science Ltd., Christchurch, New Zealand), according to the manufacturer’s protocol, in order to obtain a homogeneous population of particles primarily sized below 150 nm, as confirmed by tunable resistive pulse sensing (tRPS) technology (EXOID, Izon Science Ltd.) on NP100 membrane. Particle concentration was then standardized by using CPC100 calibration solution (110 nm mean carboxylate polystyrene beads).

For morphological analysis, a Tecnai G2 (FEI, Hillsboro, OR, USA) transmission electron microscope (TEM) operating at 100 kV was used, which confirmed that obtained nanoparticles primarily fell within “small EVs”, with a size range of 30–150 nm [40].

NDEVs were isolated from the whole EV population by an immunoaffinity capture system employing L1CAM-decorated streptavidin magnetic beads. Briefly, 100 μL of streptavidin magnetic beads (Promega Italia Srl, Milan, Italy) was decorated with 7 μg of biotinylated-anti L1CAM antibody, clone n.3H7B9 (Proteintech, Manchester, UK), according to the manufacturer’s instructions. The whole isolated EV population was resuspended in 500 μL of PBS 1X and was incubated with the capture system overnight at 4 °C. Beads with NDEVs were washed three times with PBS 1X supplemented with 0.05% Tween20 and resuspended in 500 μL of RIPA buffer (Millipore, Burlington, MA, USA) supplemented with phosphatase and protease inhibitors for protein extraction (the same volume of starting serum). After a 1h incubation period on ice followed by three freeze–thaw cycles (repetition of 30 s at −80 °C and then vortex), NDEV lysates were centrifuged at 12,000× *g* at 4 °C, and the supernatants containing the proteins were aliquoted and stored at −80 °C.

Specific ELISA assays were employed to measure both oligomeric and p129-α-synuclein levels (MBS043824 and MBS038716, respectively; MyBioSource, San Diego, CA, USA) according to the manufacturer’s instructions. Standardization was obtained by isolating NDEVs from the same volume of starting serum as well as by lysing NDEVs in the same volume of buffer (500 µL for both).

### 4.3. Statistical Analysis

IBM SPSS v29.0.1.0 software (Armonk, NY, USA) was employed to perform all statistical analyses. Differences in sex distribution were assessed using the Χ^2^ test. Shapiro–Wilk test was employed to assess normal distribution of continuous variables. Based on the distribution, differences in age at examination were assessed by an ANOVA followed by a pairwise *t*-test with Bonferroni’s correction, while differences in disease duration were assessed by the Mann–Whitney test. Differences in NDEV oligomeric and p129-α-synuclein between HC and other patient groups were assessed by ANCOVA, with sex and age as covariates, followed by Tukey’s Honestly Significant Difference (HSD) test, while for comparison between PD and ET, disease duration was also considered as covariate. ROC curves were employed to assess biomarkers’ diagnostic value, and Youden Index was used to select the cut-off, giving the maximum sum of sensitivity and specificity. Spearman’s test was applied to investigate correlations between biomarkers and demographical or clinical variables. All tests were two tailed, and a *p*-value < 0.05 was considered significant for all analyses.

## 5. Conclusions

In our study, we analyzed NDEV oligomeric and p129-α-synuclein species in patients with PD and patients with ET, demonstrating that both of these proteins may be useful for distinguishing these diseases from each other and from controls. We suggest that NDEVs oligomeric and p129-α-synuclein species might represent accessible blood-derived biomarkers to support differential diagnosis in tremulous disorders in clinical practice.

## Figures and Tables

**Table 1:** Demographic features of patients with PD, patients with ET, and HCs. Data are shown as mean ± SD.

	PD (*n* = 43)	ET (*n* = 21)	HC (*n* = 45)	*p*-Value
Sex (F/M)	13/30	7/14	25/20	0.039 ^a^
Age at examination (years)	71.4 ± 5.98 *	65.0 ± 11.43	68.4 ± 9.59	0.022 ^b^
Disease duration (years)	5.7 ± 4.03	12.6 ± 9.09	-	0.005 ^c^
MDS-UPDRS	22.3 ± 10.52	-	-	-
H&Y rating scale	2.1 ± 2.12	-	-	-
Fahn–Tolosa–Marín Tremor rating scale	-	10.6 ± 6.47	-	-

PD: Parkinson’s disease; ET: essential tremor; HC: healthy control; MDS-UPDRS: MDS—Unified Parkinson’s Disease Rating Scale; HY: Hoehn and Yahr. ^a^ Χ^2^ test. ^b^ ANOVA followed by pairwise *t*-test with Bonferroni correction. ^c^ Mann–Whitney U test. * PD vs. ET (*p*-value: 0.021).

**Table 2:** Correlation analysis of oligomeric and p-129-α-synuclein levels with demographic and clinical variables in PD and ET and with sex and age in HC.

			Sex	Age	Disease Duration	MDS- UPDRS-III	H&Y Score	Fahn–Tolosa–Marín Tremor Rating Scale
PD (*n* = 43)	o-α-syn	Spearman’s rho	0.18	−0.11	0.12	0.07	0.08	-
		*p*-value	0.26	0.50	0.46	0.65	0.60	-
	p129-α-syn	Spearman’s rho	0.10	0.18	0.20	0.08	0.10	-
		*p*-value	0.52	0.26	0.20	0.59	0.54	-
ET (*n* = 21)	o-α-syn	Spearman’s rho	0.17	−0.05	0.31	-	-	−0.16
		*p*-value	0.47	0.82	0.17	-	-	0.55
	p129-α-syn	Spearman’s rho	0.28	0.10	0.09	-	-	0.02
		*p*-value	0.21	0.68	0.70	-	-	0.94
HC (*n* = 45)	o-α-syn	Spearman’s rho	0.18	0.18	-	-	-	-
		*p*-value	0.24	0.24	-	-	-	-
	p129-α-syn	Spearman’s rho	−0.04	−0.06	-	-	-	-
		*p*-value	0.81	0.71	-	-	-	-

PD: Parkinson’s disease; ET: essential tremor; HC: healthy control; MDS-UPDRS: MDS—Unified Parkinson’s Disease Rating Scale; HY: Hoehn and Yahr; o-α-syn: oligomeric α-synuclein; p129-α-syn: 129Ser-phosphorylated α-synuclein.

## Data Availability

Due to privacy restrictions, the data supporting the results of this study are not publicly available and can be reasonably requested from the corresponding author.

## Abbreviations

The following abbreviations are used in this manuscript:

PD	Parkinson’s disease
ET	Essential tremor
HC	Healthy control
LBs	Lewy bodies
EVs	Extracellular vesicles
NDEVs	Neuronally derived extracellular vesicles
CSN	Central nervous system
MDS-UPDRS	MDS—Unified Parkinson’s Disease Rating Scale
HY	Hoehn and Yahr
ROC	Receiver operator characteristic
AUC	Area under the curve
RT-QuIC	Real-time quaking-induced conversion
